# Generalized nonlinearity in animal ecology: Research, review, and recommendations

**DOI:** 10.1002/ece3.11387

**Published:** 2024-07-11

**Authors:** David R. Heit, Waldemar Ortiz‐Calo, Mairi K. P. Poisson, Andrew R. Butler, Remington J. Moll

**Affiliations:** ^1^ Department of Natural Resources and the Environment University of New Hampshire Durham New Hampshire USA; ^2^ Wildlife Biology Program, W.A. Franke College of Forestry University of Montana Missoula Montana USA

**Keywords:** additive models, generalized linear models, general liner models, model assumptions, regression

## Abstract

Generalized linear models (GLMs) are an integral tool in ecology. Like general linear models, GLMs assume linearity, which entails a linear relationship between independent and dependent variables. However, because this assumption acts on the link rather than the natural scale in GLMs, it is more easily overlooked. We reviewed recent ecological literature to quantify the use of linearity. We then used two case studies to confront the linearity assumption via two GLMs fit to empirical data. In the first case study we compared GLMs to generalized additive models (GAMs) fit to mammal relative abundance data. In the second case study we tested for linearity in occupancy models using passerine point‐count data. We reviewed 162 studies published in the last 5 years in five leading ecology journals and found less than 15% reported testing for linearity. These studies used transformations and GAMs more often than they reported a linearity test. In the first case study, GAMs strongly out‐performed GLMs as measured by AIC in modeling relative abundance, and GAMs helped uncover nonlinear responses of carnivore species to landscape development. In the second case study, 14% of species‐specific models failed a formal statistical test for linearity. We also found that differences between linear and nonlinear (i.e., those with a transformed independent variable) model predictions were similar for some species but not for others, with implications for inference and conservation decision‐making. Our review suggests that reporting tests for linearity are rare in recent studies employing GLMs. Our case studies show how formally comparing models that allow for nonlinear relationships between the dependent and independent variables has the potential to impact inference, generate new hypotheses, and alter conservation implications. We conclude by suggesting that ecological studies report tests for linearity and use formal methods to address linearity assumption violations in GLMs.

## Graphical abstract

This paper shows that (1) linearity is often overlooked—or at least unreported—in ecological studies using Generalized Linear Models, and (2) failing to consider the linearity assumption can affect inference and hinder development of novel hypotheses. We conclude with clear recommendations for future research.

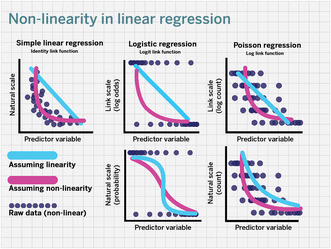

## 1 INTRODUCTION

Ecological systems are complex, and this complexity often presents a formidable statistical challenge to researchers trying to disentangle interacting variables that rarely relate to one another in a straightforward, linear manner (Anand et al., 2010; Bolker et al., 2013; Maurer, 1999; Moll et al., 2016). Consequently, the statistical methods used in ecology have rapidly developed over the last several decades, and quantitative expertise is often highly valued among ecologists, even to the point of controversy (Ellison & Dennis, 2010; McGill, 2012; Millspaugh & Gitzen, 2010). As quantitative methods continue to advance and complex models become commonplace, it can become easy to overlook foundational assumption checks (Long, 2008; Nimon, 2012; Osborne & Waters, 2019). Yet regardless of how sophisticated statistical methods become, understanding and reporting foundational assumption checks remains critical (Zuur et al., 2010).

The general linear model is a cornerstone of ecological data analysis. This model and its variations encompass traditional analyses used in experimental settings (e.g., *t*‐tests, ANOVAs) as well as simple and multiple regressions. However, ecological analyses are sometimes limited by the assumptions of general linear models, especially those requiring the independence, constant variance, and normality of errors and the linearity of the relationship between independent and dependent variables. The normality of errors is violated in many, if not most, ecological situations. For example, non‐normal errors are expected when modeling ecological responses captured by binary (i.e., zero or one) or count (i.e., non‐negative integers) data. These data types abound in nature and ecological research. Common examples of binary data include species distributions and occurrence (e.g., individuals or species present or not; Devictor et al., 2008; Guisan & Thuiller, 2005), detectability (e.g., present species or individuals detected or not; MacKenzie et al., 2002; Tyre et al., 2003), or survival and mortality (e.g., individuals alive or not; Evans et al., 2015). Count data are also common, as they quantify population abundance, species richness, and biodiversity (Bohmann et al., 2014; Dallimer et al., 2012), or any situation where individuals, species, or ecological processes are measured as discrete, countable units (e.g., predation events, reproduction attempts, site visits; Kéry & Royle, 2015). Given their ubiquity, we focus this paper on modeling situations involving these two types of dependent, or response, variables. To model the effects of independent variables on these responses, researchers typically turn to generalized linear models (GLMs).

### 1.1 Generalized linear models overview

GLMs overcome some of the limitations of general linear models by relating independent and dependent variables to each other via a mathematical function that connects a given parameter of a non‐normal distribution (typically, its mean or expected value) to a linear combination of independent variables and associated parameters (the linear predictor; Nelder & Wedderburn, 1972). This mathematical function is known as the *link function*, and the scale on which it relates the expected value of independent variable(s) to the dependent variable is the *link scale*, as opposed to the *natural scale* which is the scale of the original data (Fox et al., 2015; Gotelli & Ellison, 2004). A logit link function is commonly used to model binary dependent data via logistic regression. This link models the natural logarithm of the odds ratio as a function of the linear predictor and is often back‐transformed to interpret effects on the mean expected probability of success (i.e., obtaining a “one,” such as species presence, the state of being alive, or having a disease). The errors in this model are assumed to follow a Bernoulli distribution. For count data, a log link function is typical. This link function relates the natural logarithm of the mean expected count to the linear predictor, and the error structure is described using a Poisson distribution.

Of course, the use of link functions and non‐normal distributions does not eliminate modeling assumptions, but rather changes them from those present in general linear models. In GLMs, there remains the assumption of independent observations, or, more technically, independent errors (Bolker, 2008), and much work has focused on the causes, consequences, and solutions to non‐independent model errors (Hurlbert, 1984; Rhodes et al., 2009; Silva et al., 2022). There also remains an assumption of a mean–variance relationship, but, unlike general linear models, GLMs typically do not assume a constant variance. Rather, variance terms follow the assumptions of the error distribution employed. For example, in Poisson distributions the variance is assumed to be equal to the mean, and in Bernoulli distributions the variance is the mean times one minus the mean. Thus, in both cases, the variance changes along with the mean. Like the issue of non‐independent errors, much work has examined these mean–variance relationships and their nuances in GLMs (e.g., overdispersion, underdispersion, zero‐inflation; Kéry & Royle, 2015; Lindén & Mäntyniemi, 2011; Martin et al., 2005).

### 1.2 The linearity assumption

This paper focuses on another assumption shared between GLMs and general linear models: the linearity assumption. Linearity entails that independent variables in a model have a linear relationship with the dependent variable, such that a constant change in an independent variable leads to a constant change in the dependent variable. Mathematically, the linearity assumption is often more important than those related to error independence, variance, or structure, and breaking this assumption can induce both bias and imprecision in model estimates (Gelman et al., 2020). Note that the linearity assumption is related to the concept of additivity, and applies to the parameters of a linear model rather than the independent variables per se. Thus, a model with a transformed independent variable (e.g., using a natural logarithm) or independent variables raised to fixed powers (e.g., a squared term, which we refer to as multi‐coefficient models) are still linear models. For example, the model *y* = *β*_0_ + *β*_1_ *x* ^2^ yields a curvilinear relationship with the dependent variable on the scale of *x* and a straight‐line relationship on the scale of *x* ^2^, but is nonetheless a linear model because the response is expressed as a linear combination of its parameters (Bolker, 2008). By contrast, the model *y* = *β*_1_ *x* ^*β*2^ is a nonlinear model because the response is not a linear combination of its parameters. The term nonlinearity can often be confusingly used to describe both kinds of nonlinear relationships. In this paper, we use the term nonlinearity in both senses but make distinctions when necessary.

**FIGURE 1:**
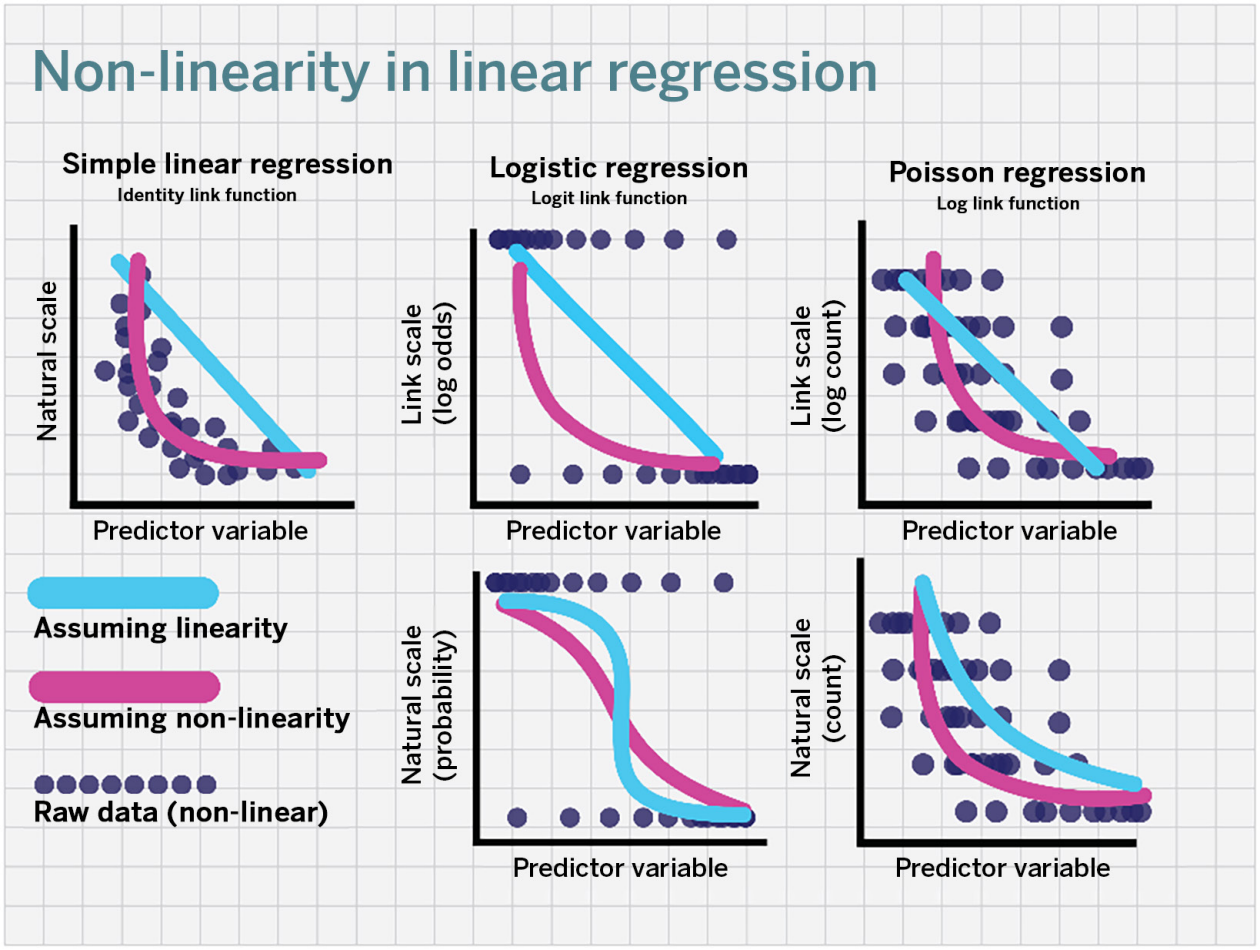
Conceptual representations of relationships in linear regression across three link functions: identity, logit, and logarithmic. Relationships shown are based on simulated relationships, where the underlying data have a nonlinear on the link scale (i.e., breaking the linearity assumption). Raw data points are shown as purple circles, and linear and nonlinear relationships as blue and magenta lines, respectively. Raw data for GLMs are the same on both the natural and link scales (binary for logistic regression, counts for Poisson regression).

GLMs share the linearity assumption with general linear models, but the assumption applies to the link rather than the natural scale (McCullagh & Nelder, 1989). This feature makes the linearity assumption more difficult to conceptualize in GLMs because model interpretation often occurs on the natural rather than the link scale, and relationships between independent and dependent variables on the natural scale are often nonlinear because of the link function (Figure 1). Put simply, a GLM might meet the linearity assumption but look nonlinear on the natural scale and vice versa. Thus, potential nonlinearity on the link scale may not be readily apparent, which might lead to a tendency to overlook or misunderstand the linearity assumption in GLMs (Boldina & Beninger, 2016; Cohen et al., 2013). One traditional diagnostic to examine the linearity assumption in GLMs is to visually inspect standardized residuals (e.g., Pearson's residuals) plotted against model predictions on the link scale (McCullagh & Nelder, 1989; Pierce & Schafer, 1986). This approach, however, relies on a subjective assessment of linearity, and as we will see in Case Study 1 below, might not always reveal meaningful or conspicuous deviations from the linearity assumption.

### 1.3 Addressing nonlinearity

Various strategies can be employed when the linearity assumption is violated. One simple strategy is to transform or alter one or more independent variables, as in the above example where the “*x*” variable is squared (Gelman et al., 2020; McCullagh & Nelder, 1989). Another common transformation is to take the logarithm of one or more independent variables. Logarithmic transformations are common when an independent variable varies by orders of magnitude and its effect on the dependent variable is expected to be nonlinear (e.g., as with body mass variables in studies of allometric scaling; Brown & Maurer, 1989; Kerkhoff & Enquist, 2009). Note that if a log transformation is performed within a GLM, it results in a nonlinear (i.e., not a straight line) relationship for body mass with the mean of the response variable on the natural scale, but a linear relationship with *log* of body mass on the link scale; such transformations obviously necessitate care when interpreting GLM model parameters and making inferences. Any such transformations also affect the mean–variance relationship of the model. Although such effects are an important topic, we focus this paper on the linearity assumption rather than mean–variance changes and refer readers to other recent works examining them (Warton, 2018; Warton & Hui, 2011).

An alternative to the transformations above is the use of generalized additive models (GAMs) that allow for complex relationships by fitting smoothing functions to one or more variables (Wood, 2017). GAMs use combinations of additive polynomials (i.e., smoothed functions) in the linear predictor, which allows for “wiggliness” in a fitted relationship that can conform to complex patterns (Pedersen et al., 2019; Wood, 2017). These individual polynomial terms (often called “splines” or “knots”) determine a model's flexibility or “wiggliness” (Pedersen et al., 2019; Wood, 2017). GAMs somewhat blur the lines between linear and nonlinear models. Technically, they are linear models, but because they fit smoothed functions in the linear predictor, they can result in almost any nonlinear relationship between the dependent variable and independent variables. Also note that GAMs fit to binary or count response data still typically require link functions and Bernoulli or Poisson distributional error structures to realistically represent ecological processes. Thus, in such cases, GAMs can be viewed as an extension of GLMs. Such models are useful in situations where nonlinear relationships between dependent and independent variables are expected but there is not strong a priori reason to limit these relationships to a particular nonlinear curve. For example, Montgomery et al. (2018) hypothesized that heat‐sensitive moose (*Alces alces*) might cope with elevated summer ambient temperatures in a threshold or other, more complex nonlinear manner and used GAMs fit to moose movement data to quantify this relationship.

Regardless of the strategy used to address linearity violations in GLMs, ultimately a researcher must decide upon a final model (or model set) for inference. While a full treatment of model selection is beyond the scope of this paper, we mention two approaches here to provide context for the case studies below. One common model selection method is the use of a selection criterion based in information theory, with AIC being by far the most popular choice for models analyzed in a frequentist or maximum likelihood framework (Akaike, 1974; Moll et al., 2016). Briefly, AIC formally trades off model fit (likelihood) with model complexity (number of parameters), thereby helping a researcher arrive at a “parsimonious” model for inference that balances the bias‐variance tradeoff (Burnham & Anderson, 2002). In the Bayesian paradigm, model selection is less straightforward, and recent work has compared and debated approaches (Hooten & Hobbs, 2015; Tenan et al., 2014; Ward, 2008). A simpler alternative to model selection using information criteria, whether in a frequentist or Bayesian paradigm, is a hypothesis‐testing approach where the decision to keep a transformation of an independent variable is based upon that transformed variable's statistical significance using cutoff values (e.g., when a parameter's 95% confidence or credible intervals fails to overlap zero; Gelman et al., 2020; McCullagh & Nelder, 1989). Such decision rules can be useful when deciding between various representations of a particular independent variable of interest within complex, hierarchical models (e.g., a conservation‐relevant landscape variable affecting species abundance or occurrence; Benítez‐López et al., 2010; Ktitorov et al., 2008; Palomino & Carrascal, 2007).

Here, we investigate the issues described above in the context of the two canonical GLMs in ecological research: Poisson regression and hierarchical binary logistic regression (i.e., occupancy models). Although the importance of the linearity assumption is not a new concept (see, e.g., Sheppard, 1914), a cursory survey of the literature and our own experience discussing models with ecologists suggested that its importance is perhaps overlooked in light of modern GLM applications to complex ecological data. We therefore used a formal literature review and two case studies to pursue three specific objectives: (1) to determine the frequency at which animal ecologists report testing for linearity or adapting models to accommodate nonlinearity (in the broad sense), (2) to explore the dynamics of linearity assumptions on the link scale of GLMs and the consequences of not addressing it, and (3) to provide reflections on lessons learned in the above process to help ecologists more clearly understand the linearity assumption and its implications for GLMs.

## 2 LINEARITY REVIEW

### 2.1 Methods

We conducted a formal literature review to determine the degree to which researchers reported testing for linearity or used “nonlinear” modeling approaches, defined here as those that allow for a nonlinear relationship between independent and dependent variables on the link scale (e.g., through a transformation, GAM, etc.). We queried all studies published during a 5‐year period (2018–2022) in five leading ecology journals (*Methods in Ecology and Evolution*, *Ecography*, *Ecology Letters*, *Ecology*, and the *Journal of Animal Ecology*). We searched for studies that contained in their title or abstract the terms “model” or “regression,” and the terms “wildlife” or “animal.” From these, we retained studies for analysis that we could confirm (1) were the result of original research; and (2) analyzed data using a general linear regression, GLM, or GAM. This process excluded papers that introduced new R packages, described conceptual models or models made for one specific purpose that were not regressions, or used “black box” methods for which model specifications were seldom described, such as hidden Markov models and machine learning algorithms. For each relevant study, we recorded: (1) if the study reported a test for linearity (on the link scale); (2) if the study used a transformation of an independent variable (e.g., a logarithmic transformation); (3) if the study used a multi‐coefficient model, defined as models that had more than one coefficient associated with an independent variable (e.g., a quadratic model or GAM); and (4) the link function(s) used in the study (identity, logit, or log).

### 2.2 Results

**FIGURE 2:**
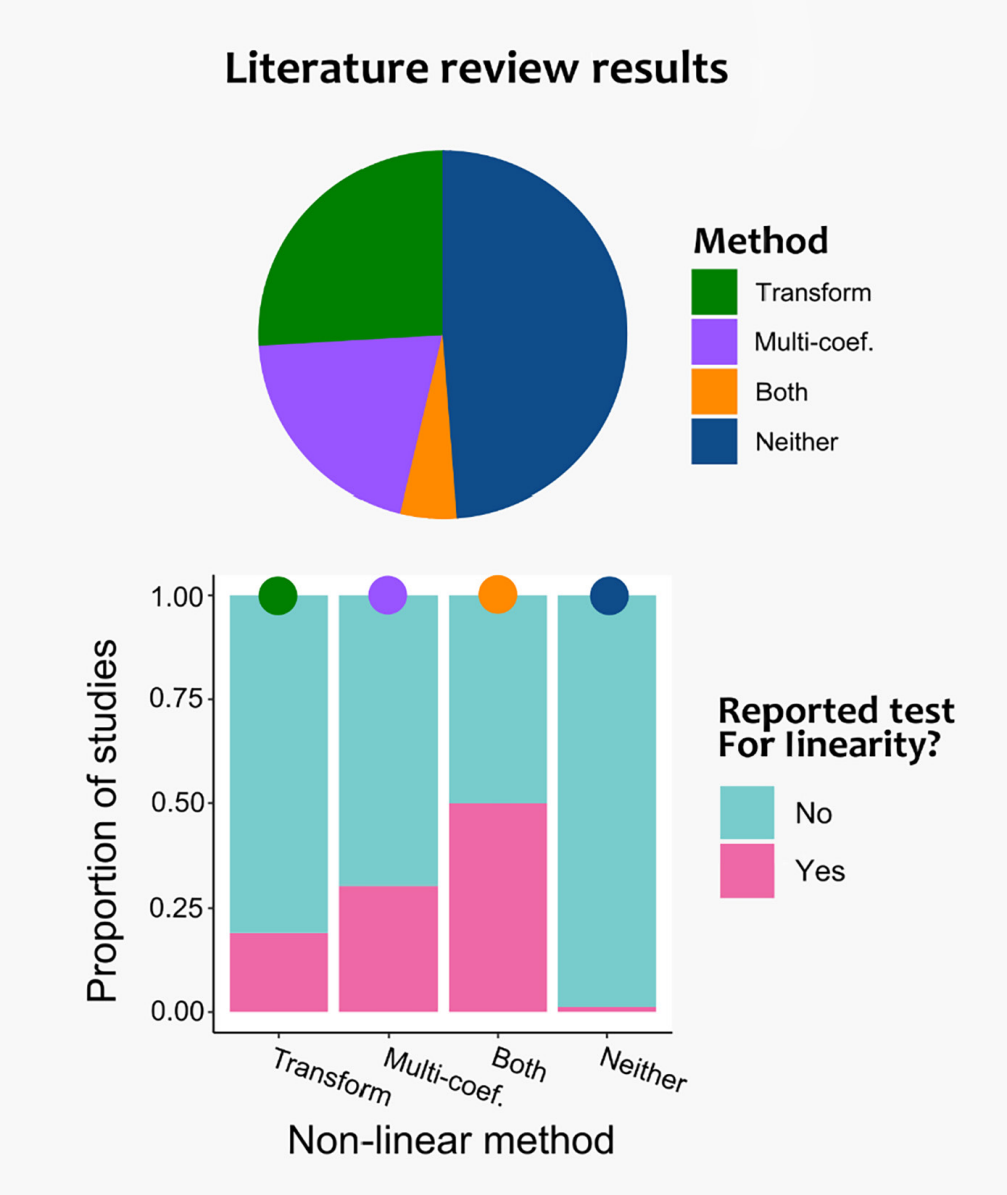
Results of a literature review of studies published between 2018 and 2022 in five leading ecological journals. The pie chart shows the proportion of studies (out of a total of 162) that included transformations of independent variables, multi‐coefficient models (abbreviated “multi‐coef,” in figure; these included models with more than one coefficient for an independent variable), both, or neither. The bar chart shows how often a study also reported a test for linearity within the proportions from the pie chart so that proportions sum to 1.0.

We reviewed 273 studies, of which 162 met the criteria described above for analysis. We found that 14.2% (*n* = 23) of studies reported testing for nonlinearity, 25.9% (*n* = 42) of studies transformed an independent variable, 20.4% (*n* = 33) of studies fit a multi‐coefficient model, 4.9% (*n* = 8) of studies used both a transformation and a multi‐coefficient model, and 48.8% (*n* = 79) used neither (Figure 2). Additionally, 33.3% (*n* = 54) of studies used an identity link function, 40.1% (*n* = 65) used a logit link function, and 34.6.% (*n* = 56) used a log link function (note that some studies used multiple link functions and multiple methods, thus proportions exceed 100%). Within studies that used an identity link function 19% (*n* = 10) reported testing for linearity, 48% (*n* = 26) used a transformation, and 17% (*n* = 9) used a multi‐coefficient model. Within studies that used a logit link function 15% (*n* = 10) reported testing for linearity, 26% (*n* = 17) used a transformation, and 26% (*n* = 17) used a multi‐coefficient model. Within studies that used a log link function 7% (*n* = 4) reported testing for linearity, 21% (*n* = 12) used a transformation, and 14% used a multi‐coefficient model. Within each of the methods to meet the linearity assumption, the proportion of studies that reported testing for linearity were: 19.1% (*n* = 8 of 42) of studies using transformations, 30.3% (*n* = 10 of 33) of studies using multi‐coefficient models, 50% (*n* = 4 of 8) of studies using both, and 1.3% (*n* = 1 of 79) of studies using neither.

### 2.3 Discussion

In our review of contemporary animal ecology literature, studies seldom reported using linearity tests before applying a “nonlinear” technique. For instance, more than 25% of studies used a transformation in their analysis, but less than a quarter of those studies reported testing for linearity. This pattern persisted across each of the link functions we evaluated (identity, logit, and log), and testing for linearity was less common in studies that used GLMs than general linear regression. Overall, less than 15% of studies reported testing for linearity. This trend could be an indication of multiple dynamics occurring separately or concurrently. Studies could be applying nonlinear methods (i.e., transformations, GAMs) more often than they test for linearity, basing their decision either on a priori expectations or prior research. Conversely, studies could be testing for linearity, but not including those results in the main text. Both scenarios suggest that explicitly testing the linearity assumption and reporting the results of such tests is uncommon in recent animal ecology literature. There are specific variables in ecology research where “nonlinear” techniques are almost universally applied and explaining their use is likely redundant, such as log‐transformations of body mass in allometry research (Kerkhoff & Enquist, 2009). Nonetheless, the nuances of transformations on the link scale of GLMs are still worth reporting even for such variables, and our findings suggest that there might be a lack of attention placed on linearity assumptions for other GLM modeling situations.

## 3 CASE STUDY 1: MODELING MAMMAL ABUNDANCE USING POISSON REGRESSION

### 3.1 Background and study design

This case study focuses on the relative abundance modeling of coyotes (*Canis latrans*) and red foxes (*Vulpes vulpes*, hereafter “foxes”). These two canid species are competitors, and coyotes are dominant over foxes and can reduce their abundance through interference competition and intraguild predation (Gosselink et al., 2007; Levi & Wilmers, 2012; Moll et al., 2018). Prior research on these species suggests that their competitive interactions might be mediated by human development of the landscape (Moll et al., 2018, 2023). Specifically, a “human shield” effect might reduce the interference competition experienced by foxes from coyotes, which are more human averse and therefore less abundant in more developed locations that foxes readily inhabit (Moll et al., 2018). However, this relationship does not always cleanly manifest (Cervantes et al., 2023; Mueller et al., 2018), suggesting that a nonlinear relationship among these variables could be present. Thus, these species act as a useful case study to explore nonlinearity in a GLM framework by extending a traditional GLM to a GAM.

**FIGURE 3:**
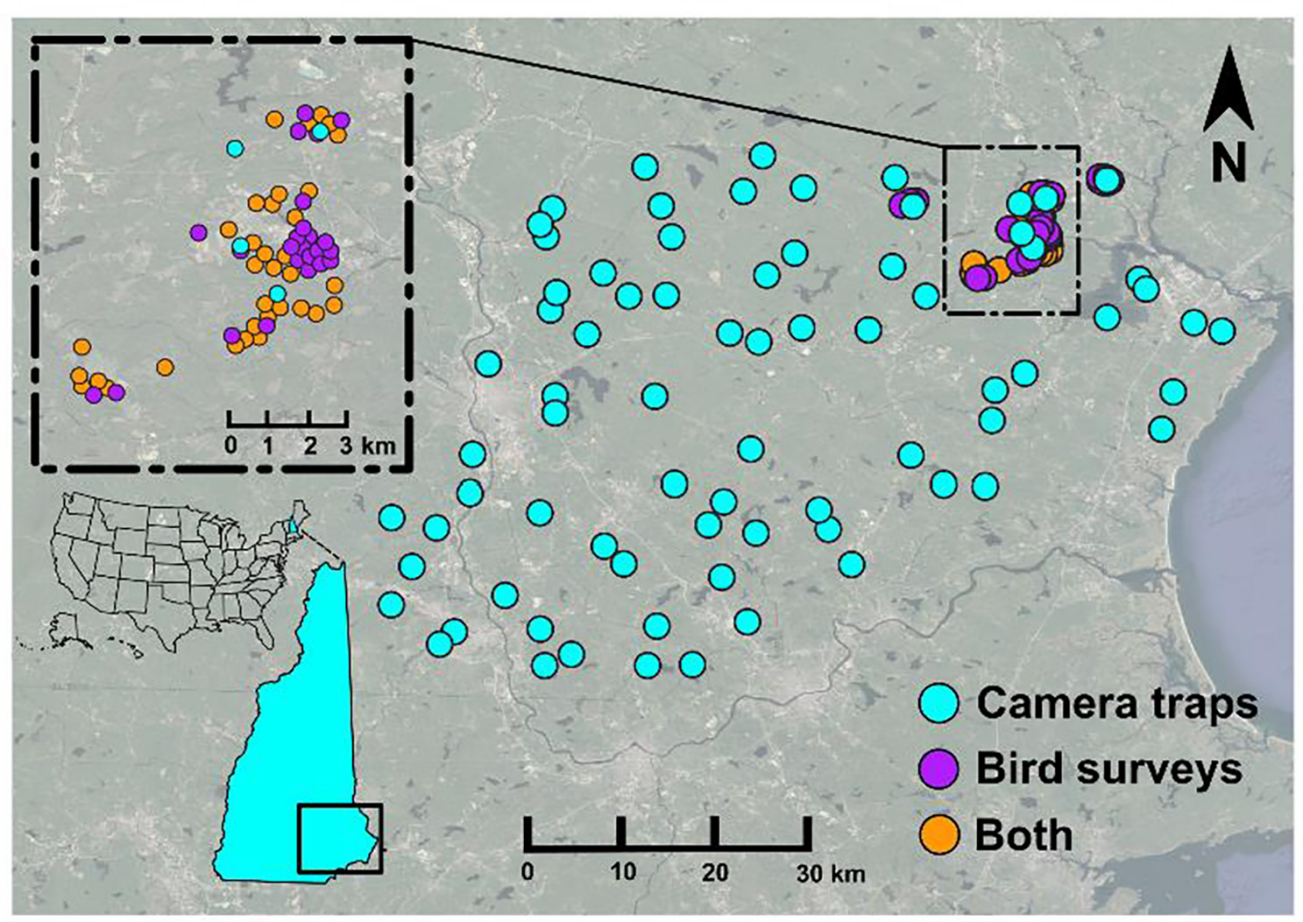
Study area for two case studies in Southeastern New Hampshire, USA. The first case study (blue and orange circles) used data from an array of 109 camera traps deployed in summer of 2021. The second case study (purple and orange circles) used point‐count survey data from passerines on lands managed by the University of New Hampshire in the summers of 2021 and 2022.

To quantify relative abundance, we deployed camera traps across 109 sites in the 3200 km^2^ Southeast Management Region in New Hampshire, USA (New Hampshire Fish and Game [NHFS], 2022), 34 of which coincided with locations also used for bird point counts in case study 2 (Figure 3). The study area consists of approximately 49% forested land, 25% anthropogenic development, 14% wetland, and 4% open water. The remaining 8% is comprised of a combination of agriculture, grasslands, shrublands, and sand beaches (Dewitz & U.S. Geological Survey, 2021). We selected 75 of the camera sites using a generalized random tessellation design with sites separated by at least 1 km (Stevens & Olsen, 2003), and we selected the remaining 33 sites using a fully random design within properties managed by the University of New Hampshire with sites separated by at least 250 m (Moll et al., 2023; Poisson et al., 2023). We selected sites using QGIS (QGIS Association, 2021), then made minor adjustments in the field to locate a suitable location to affix the camera on a tree at approximately 50 cm above ground, facing north, and not conspicuously placed to avoid tampering. Cameras took three images with each motion trigger, and then did not re‐trigger for 5 min (Lepard et al., 2018). For this case study, we used images captured during summer 2022 (June 15–September 15).

### 3.2 Abundance modeling

We modeled the relative abundance of these mammal species using a Poisson regression with the number of days with at least one detection as the dependent variable. For each species we fit two models: a Poisson GLM, and a Poisson GAM. In each GLM and GAM we included three independent variables: road density within 1 km^2^ (a proxy for urban development in this study area), distance to nearest water source, and distance to nearest agricultural area. We included the latter two non‐urban variables based on their importance to these focal species in previous work (Moll et al., 2023). We obtained road data from the TIGER county‐level road database (U.S. Census Bureau, 2019). We derived all other variables using the 2019 National Land Cover Database (Dewitz & U.S. Geological Survey, 2021). In GAMs, we used restricted maximum likelihood to define our smoothing functions and thin‐plate regression to determine the number of splines but constrained the smoothing basis functions to 10 dimensions (*k* = 10) to encompass the estimated degrees of freedom for all species (Wood, 2017). To evaluate whether the GAMs provided a better model fit than the GLMs, we compared GLMs to GAMs using the Akaike Information Criterion (AIC). For the GLMs, we also visually inspected plots of Pearson residuals versus model predictions on the link scale. For model visualizations, we fit univariate models of each independent variable and each species.

### 3.3 Results

**FIGURE 4:**
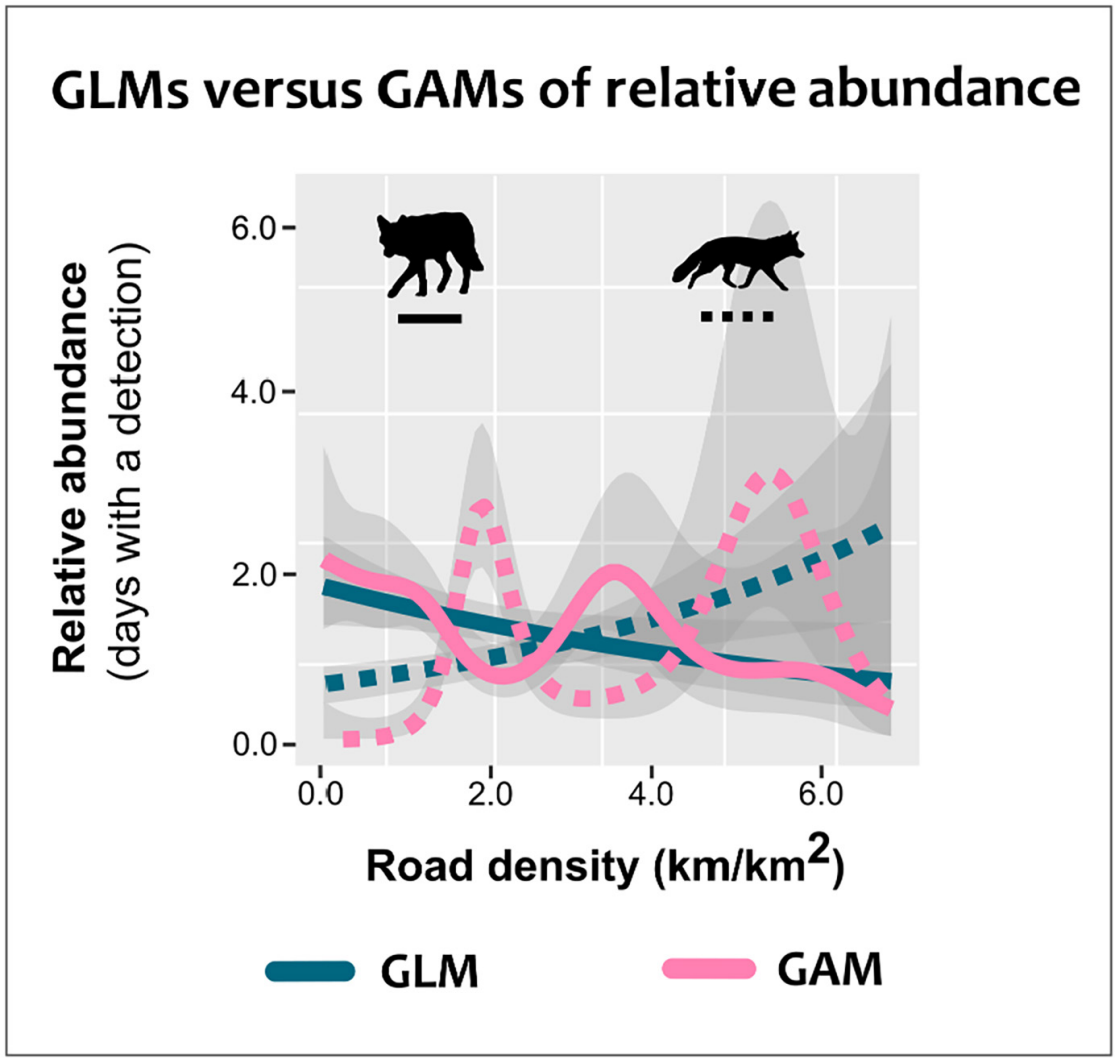
Generalized linear models (GLMs) and generalized additive models (GAMs) of relative abundance fit to camera trap data of coyotes (*Canis latrans*) and red foxes (*Vulpes vulpes*) collected in the summer of 2021 in New Hampshire, USA.

We recorded 354 images (*n* = 203 coyote and *n* = 151 foxes) between June 15 and September 15, 2022. Visual inspection of residuals versus model predictions in the GLM did not strongly suggest nonlinearity on the link scale for either species (Figure S1, Data S1). Nonetheless, given that a difference of 10 or more AIC units represents a substantial change support between models (Burnham & Anderson, 2002), the GAMs for both species were strongly favored over regular GLMs by AIC (coyote ΔAIC = 32.8, fox ΔAIC = 214.5; Table 1). We found that the GLMs for foxes and coyotes were approximately the inverse of each other for road density, with coyotes having a negative relationship with road density and foxes having a positive relationship. In the GAMs, however, we found this relationship to still be inversed, but in a more nuanced nonlinear pattern where peaks in coyote relative abundance coincided with troughs in fox abundance and vice‐versa (Figure 4).

**TABLE 1:** Comparisons between generalized linear model (GLM) and generalize additive model (GAM) specifications of coyotes (*Canis latrans*), and red foxes (*Vulpes vulpes*).

GLM							
Species	Road density		Distance to water		Distance to agriculture		AIC
	Estimate	*p*‐Value	Estimate	*p*‐Value	Estimate	*p*‐Value	
Coyote	−0.18	.064	0.08	.299	−0.29	**.002**	457.8
Red fox	0.31	**<.001**	0.08	.418	−0.12	**.0261**	509.4

GAM							
Species	Road density		Distance to water		Distance to agriculture		AIC
	EDF	*p*‐Value	EDF	*p*‐Value	EDF	*p*‐Value	
Coyote	4.583	**.006**	4.645	**<.001**	1.001	**<.001**	425.0
Red fox	6.733	**<.001**	6.260	**<.001**	5.749	**<.001**	294.9

*Note*: Data for the models was obtained from camera traps in New Hampshire, USA. Independent variables for both model types are: Road density within 1 km, distance to nearest water source, and distance to nearest agricultural area. Significant *p*‐values (<.05) are bolded.

### 3.4 Discussion

We found that GAMs not only outperformed GLMs in Poisson regression of relative abundance but also elucidated patterns not present in the GLMs. Such patterns provide additional ecological interpretations that would not be able to be drawn from the GLMs alone. When inspecting only the GLM, we might deduce that coyotes and foxes simply had differing tolerances to anthropogenic development. However, the GAM revealed a more complex nonlinear pattern where red fox abundance peaked at moderate levels of development coincident with the lowest estimated coyote abundance (Figure 4). This pattern could be the result of foxes interfacing with anthropogenic development primarily to avoid coyotes spatially, that is, a “human shield effect” (Moll et al., 2018). This nonlinear relationship was only recognizable when using a GAM, and no output of the GLM necessarily pointed to the necessity of fitting a GAM, including visual inspection of residual plots. Note that for both approaches (GLM and GAM), there is high uncertainty around the mean predictions at higher road densities (Figure 4). This uncertainty is partly a result of fewer sites with high road density (Figure S2). Thus, in the case of the GAM, the pattern of a second peak in fox abundance at ~5 km/km^2^ road density warrants further investigation to determine if this was merely a feature of a small number of sites in this study area or whether foxes truly do show higher abundances in such sites due to other factors (e.g., higher prey availability due to greater edge habitat). Additionally, the GAM predicted low abundance for both coyote and red fox at the highest road densities, whereas the GLM predicted the highest fox abundance at the highest road density.

Interpreting the numerical outputs of a GAM may be less straightforward than a standard GLM that has one coefficient for each independent variable. The multiple coefficients that result in GAMs are potentially not as easily communicable or actionable for practitioner stakeholders like land managers. However, the utility of GAMs, as we found in this case study, is often in the visual representation of relationships, which can elucidate dynamics absent in linear models or GLMs. Such dynamics might be interpreted directly or could suggest novel hypotheses to be explored in future work. However, with GAMs' flexibility comes sensitivity to input parameters like smoothing basis functions and the number of splines to use; as such, misspecifications can lead to over or underfitting (Furey et al., 2013; Pedersen et al., 2019; Wood, 2017). It is not sufficient to fit a GAM with default specifications without inspection of data properties like estimated degrees of freedom and assume that nonlinearities have been adequately captured. GAMs frequently require fine‐tuning to achieve an appropriate fit (see Wood, 2017 for details). Therefore, it is important to be thorough in preliminary evaluations of one's data, specify the parameters of GAMs accordingly, and justify the choices made (see Pedersen et al., 2019; Wood, 2017).

## 4 CASE STUDY 2: MODELING PASSERINE OCCUPANCY

### 4.1 Background and study design

In this case study, we examine a passerine community (i.e., songbirds, order *Passeriformes*) in Southeastern New Hampshire. This community includes several dozen species, and some species‐specific habitat requirements could be nonlinear, especially for landscape variables like land cover type and anthropogenic development (Benítez‐López et al., 2010; Ktitorov et al., 2008; Palomino & Carrascal, 2007).

We conducted independent‐observer point counts (Frey et al., 2012) of passerine bird species across 70 sites in southeastern New Hampshire, USA, during the two consecutive summer breeding seasons of June–July during 2021 and 2022. We selected sites randomly within areas with land access permission and we separated sites by a minimum of 250 m. The survey period each day began 30 min before sunrise and concluded four and a half hours after sunrise, as detection rates are expected to decline beyond this time because birds reduce singing (Leu et al., 2017). Each survey lasted 8 min, during which the observer recorded all bird species seen or heard. We visited most sites three times each year (range per site across years combined = 2–6, mean = 5.36), with 379 total survey‐days.

### 4.2 Occupancy modeling

We fit occupancy models to these data, and formally tested each species for linearity on the logit link scale. As noted above, residuals versus fitted plots can provide a general evaluation of the linearity assumption and such plots can be visually inspected for each species of interest. However, for speciose communities, it is less tractable to perform this subjective analysis with each individual species. Further, for hierarchical models with latent variables such as occupancy models, the appropriate residuals to use in such visualization are somewhat unclear. Thus, we utilized an empirical test, the Box‐Tidwell test, to test for linearity assumption violations on the link scale. The Box‐Tidwell test (Box & Tidwell, 1962) fits a linear specification of a model alongside an interaction between the independent variable and the natural log of that same variable. If the interaction coefficient is significantly different from zero (typically using a cutoff of *p* < .05), it is evident that the linearity assumption is violated and indicates the presence of a nonlinear relationship on the link scale, although the test itself does not indicate the exact specifications or polynomial that should be used to accommodate this nonlinearity.

We modeled occupancy for all species with 10 or more observations across both survey years. We fit an occupancy model to each species with several independent variables hypothesized to influence these species' occurrence as well as an interaction between the variables and their natural logarithms (i.e., the Box‐Tidwell test). The independent variables included distance to nearest road, distance to nearest body of water, proportion forest cover, and proportion wetland cover, as these have potential to impact passerine occurrence in our study area (Ascensão et al., 2022; Brown et al., 2014; Rahlin et al., 2022). We also fit a detection sub‐model with time of day as an independent variable to control for heterogeneity in detection probability due to temporally variant singing behavior. For all variables we used a logistic prior distribution with a location of zero and a scale of one (Northrup & Gerber, 2018).

We considered a Box‐Tidwell test to fail when the 95% credible interval of the coefficient of the log‐transformed interaction term failed to overlap zero. For the species that failed the Box‐Tidwell test (indicating a violation of the linearity assumption), we fit univariate occupancy models with only the variable for which the species failed and compared that model to an identical one with a natural log transformation of that variable, though as we note below other polynomials could be used. We fit models in a Bayesian framework using JAGS via the “r2jags” package in R (Version 4.2.1, Plummer, 2003; R Core Team, 2022; Su et al., 2015). We fit all models with 8000 MCMC iterations with three un‐thinned chains and a burn‐in of 1000 iterations (Link & Eaton, 2012) and assured convergence using standard diagnostics.

### 4.3 Results

**FIGURE 5:**
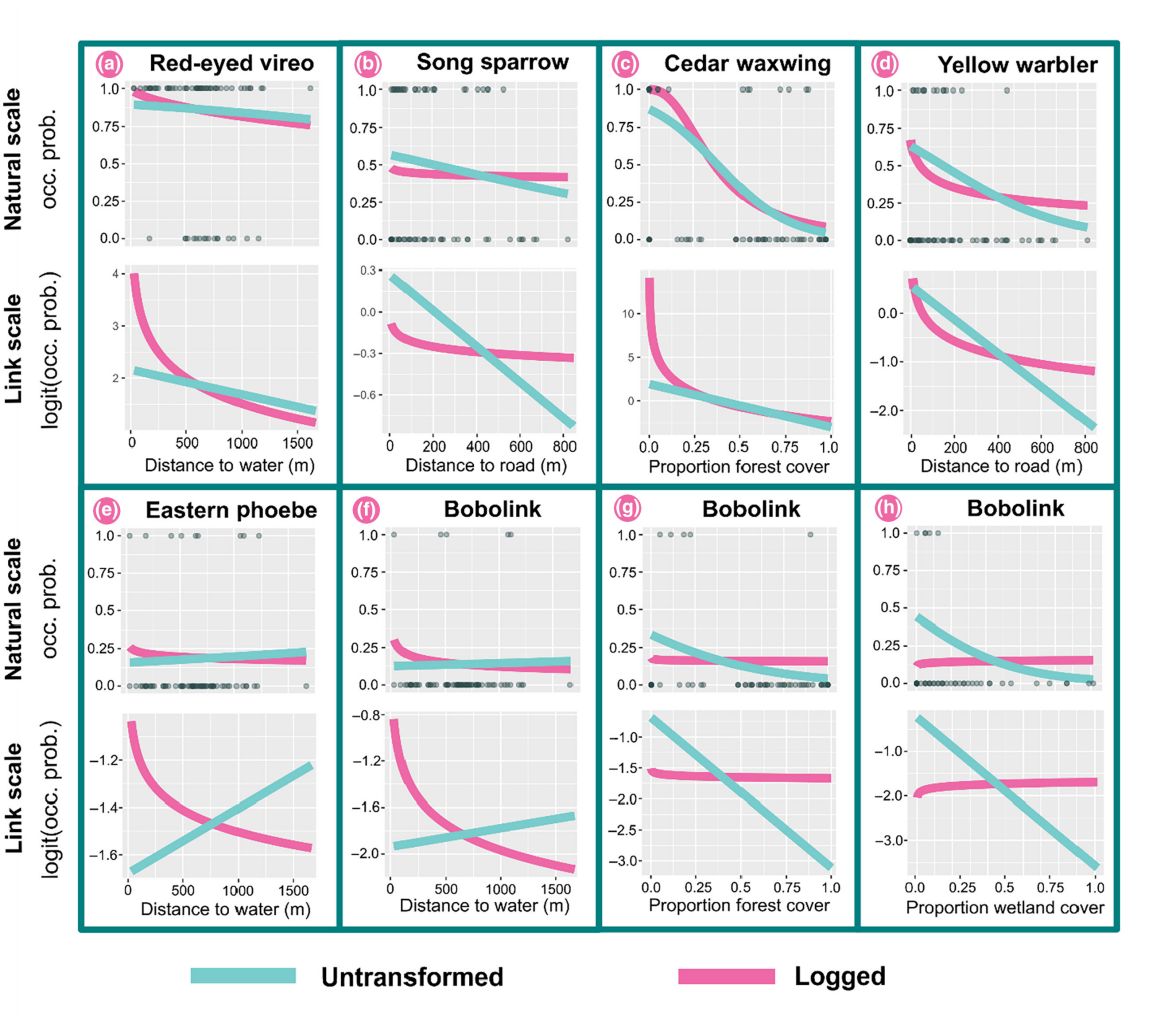
Linear and nonlinear specifications of occupancy models for passerine bird species from point count data collected in the summers of 2021 and 2022 in New Hampshire, USA. Included in the plot are those that failed a Box‐Tidwell test for linearity. Confidence intervals were omitted for clarity. Panels a‐h depict modeled predictions by species on both the natural and link scale.

We ran Box‐Tidwell tests for the five independent variables listed above for 42 bird species (a total of 210 tests). In total there were eight failures (3.8% of 210) in six species (14.2% of 42, Table 2). We observed at least one failure for each independent variable considered in the occupancy sub‐model, but none for time of day in the detection sub‐model. We also observed failures across models for species that strongly varied in commonness, as our most‐observed (red‐eyed vireo *Viero olivaceus*, *n* = 143 detections) and least‐observed species (bobolink *Dolichonyx oryzivorous*, *n* = 10 detections) both failed the Box‐Tidwell test (Table 2). In most failures, the magnitude of the effect of the independent variable was small on the natural scale (Figure 5). However, there were species that had similar trends on the natural and link scales (Figure 5, yellow warbler) and species that differed strongly between these scales (Figure 5, cedar waxwing *Bombycilla cedrorum*). In three instances, the log‐transformation yielded a positive effect where the linear case of the model was negative, or vice versa.

**TABLE 2:** Passerine bird species that failed a Box‐Tidwell test for linearity and their respective number of occurrences and variables for which the test failed.

Species	No. detections	Box‐Tidwell failure	Mean (95% CI)
Red‐eyed vireo (*Vireo olivaceus*)	143	Distance to road	2.21 (0.19, 5.35)
Song sparrow (*Melospiza melodia*)	79	Distance to road	−1.49 (−2.96, −0.32)
Cedar waxwing (*Bombycilla cedrorum*)	42	Forest cover	1.68 (0.17, 4.00)
Yellow warbler (*Setophaga petechia*)	31	Distance to road	−1.55 (−3.30, −0.09)
Eastern phoebe (*Sayornis phoebe*)	18	Distance to water	1.23 (0.14, 3.07)
Bobolink (*Dolichonyx oryzivorous*)	10	Wetland cover	−2.63 (−5.97, −0.26)
		Distance to water	1.05 (0.13–2.20)
		Forest cover	−2.10 (−5.29, −0.32)

*Note*: Results are based on models fit to a point count data collected in the summers of 2021 and 2022 in New Hampshire, USA.

### 4.4 Discussion

We found that 14% of passerine bird species failed a Box‐Tidwell test for linearity. Although this comprised a small number of the total tests we performed, the nonlinear models (i.e., via a natural logarithm transformation) we fit for the species that failed highlight the potential inferential impacts of nonlinearity on the link scale in GLMs. Firstly, for three species we found that the coefficient from the log‐transformed model was of the opposite direction than the linear specification (Figure 5e,f,h), meaning that unaddressed nonlinearity could lead researchers to opposite and possibly incorrect conclusions. Secondly, though often subtle, the differences between the linear and nonlinear GLMs are non‐trivial for ecological interpretation. For yellow warblers in this case study, the model with the logged variable predicted that the probability of occupancy would be approximately 12% less than the linear model at values of distance to nearest road near 200 m, but 15% greater near 800 m (Figure 5d). More generally, the logged yellow warbler model indicates a threshold relationship between occupancy and road proximity whereas the unlogged model indicates a more continuous decline in occupancy at locations further from roads. These differences could constitute important ecological or conservation‐relevant information. For example, the rapid decline in yellow warbler occupancy as a function of road proximity in the logged model suggests that roadside habitat is strongly preferred or perhaps even critical for this species, whereas the unlogged model indicates a weaker and more gradual relationship. The logged model relationship is intriguing in light of yellow warbler's unusual ability to cope with high levels of brood parasitism by brown‐headed cowbirds (*Molothrus ater*), another species that shows an affinity for open habitat near roads (Briskie et al., 1990; Chace et al., 2003). While the yellow warbler is not a species of particular conservation concern, this type of model inaccuracy could arise for any species and would be especially important to consider for threatened and endangered species or situations where financial resources are limited (Bottrill et al., 2009; Game et al., 2013). In such cases, accurately identifying threshold relationships is critical to both species conservation and cost‐effective decision‐making.

Finally, interpretations of GLMs typically occur on the natural scale. For some species in this case study, the natural scale and link scale relationships were quite similar (Figure 5d), but for other species with effect sizes greater in magnitude, the differences between natural scale and link scale were more pronounced (Figure 5c). Since the data in a GLM with a log‐transformed independent variable are transformed in two ways (the natural log in the linear predictor and the link function itself), the direction and magnitude of the effect on the link scale can influence how the pattern manifests on the natural scale. These observations underscore the importance of careful interpretation of model results aided by visualizations, a strategy that is useful for simple models and indispensable for more complex, hierarchical models (Spake et al., 2023). Finally, we note that this case study differs from the one above in that it was more exploratory in testing log transformations for every independent variable for each species. Such exploratory or all‐possibilities approaches should be followed with confirmatory work to minimize spurious conclusions.

## 5 CONCLUSIONS AND RECOMMENDATIONS

Generalized linear models are an integral tool in animal ecology. Like general linear models, GLMs are subject to foundational assumptions, including the assumption of linearity. This assumption applies to the link scale of the GLM, but interpretation of the outputs of GLM most often occurs on the natural scale. It is this dynamic that makes conceptualization of what a nonlinear relationship on the link scale means on the natural scale less obvious (Figure 1). Here, we demonstrated that linearity tests are rarely reported in published literature, that nonlinearity can be present on the link scale of GLMs, and that model modifications to accommodate this nonlinearity can elucidate ecological patterns obscured by standard GLMs. Accommodating this nonlinearity can not only improve model fit and improved ecological understanding, it can also provide more accurate information to guide conservation and management decisions. More broadly, ecological research would benefit additional reporting around the linearity assumption in generalized linear modeling. We note that our review was not able to distinguish between a lack of reporting and a lack of testing, nor did it systematically examine the potential consequences of either situation for the studies reviewed. We therefore do not intend to criticize this body of work, but rather use this review to highlight the nuances of the linearity assumption in GLMs and the importance of assumption check reporting. Based on these results, we describe three recommendations below. We also note that our analysis did not examine the effects of transformations of independent variables (e.g., using the natural logarithm or fitting multi‐coefficient models) and the mean–variance assumptions of various distributions; we consider this an important topic for future work.

First, we suggest that researchers considering GLMs use the raw data to visually examine the relationships between independent variables and dependent variables on both the natural and link scales as well as plots of residuals versus model predictions of fitted GLMs. While these visualizations are subjective to interpretation and thus should not be the only tool to diagnose nonlinearity and check other model assumptions, we believe that the exercise of viewing data on both natural and link scales would help researchers build intuition for what the linearity assumption entails in GLMs. Specifically, this exercise clarifies that using a link function implicitly introduces a nonlinear interpretation between the dependent variable and independent variables on the natural scale, but that does not necessarily mean that the linearity assumption is violated or unnecessary.

Second, we suggest both formally testing the linearity assumption and explicitly reporting the results of those tests, preferably in the text of a manuscript but at minimum in an appendix. Here, we use the term “test” in a general sense to include traditional statistical tests like the Box‐Tidwell test as well as formal comparisons between model specifications using model selection tools like AIC. Although this suggestion is straightforward, our literature review reveals that it is rarely performed or reported. Clear testing and reporting are particularly important in the context of GLMs to ensure that tests have been conducted on the link scale because that is the scale on which the linearity assumption acts. It is critical to note, however, that model selection tools like AIC do not typically constitute a formal test for the foundational assumptions in linear modeling. For example, a model with a lower AIC does not necessarily meet the linearity assumption more adequately than a competing model with a higher AIC. Thus, model selection tools are useful in determining if a nonlinear model formulation is supported by the data but are not strictly a substitute for model assumption checks.

Third, we suggest that the linearity tests described above and the model specifications they inspire be explicitly justified and demarcated as a priori or post hoc and/or exploratory, where a priori tests are conducted during the first formal analysis based upon hypotheses or expected relationships grounded in theory or previous work (as in Case Study 1) and post hoc tests are based upon secondary or later analyses following an initial or exploratory model analysis (as in Case Study 2). The a priori versus post hoc distinction is important to guard against spurious nonlinear relationships that are more likely to arise when many post hoc model modifications are performed (Burnham & Anderson, 2002). At the same time, post hoc tests may be highly valuable for improving model robustness by ensuring that the linearity assumption is reasonably met and for generating new hypotheses to be tested in future work.

## AUTHOR CONTRIBUTIONS

**David R. Heit:** Conceptualization (equal); data curation (equal); formal analysis (lead); funding acquisition (supporting); investigation (equal); visualization (lead); writing – original draft (lead); writing – review and editing (equal). **Waldemar Ortiz‐Calo:** Conceptualization (supporting); formal analysis (supporting); investigation (equal); writing – review and editing (equal). **Mairi K. P. Poisson:** Data curation (equal); formal analysis (supporting); investigation (supporting); writing – review and editing (equal). **Andrew R. Butler:** Data curation (equal); formal analysis (supporting); investigation (supporting); writing – review and editing (equal). **Remington J. Moll:** Conceptualization (equal); data curation (supporting); formal analysis (equal); funding acquisition (equal); investigation (equal); methodology (equal); project administration (lead); visualization (supporting); writing – review and editing (equal).

## FUNDING INFORMATION

DRH was supported by the National Science Foundation Graduate Research Fellowship Program. Funding for the empirical case studies was provided via the Pittman‐Robertson Federal Aid in Wildlife Restoration Act Grant No. F21AF01526 (W‐112‐R‐1), and the New Hampshire Fish and Game Department. Partial funding was also provided by the New Hampshire Agricultural Experiment Station. This work was supported by the United States Department of Agriculture National Institute of Food and Agriculture Hatch Project 1024128. This is the scientific contribution number 3014.

## CONFLICT OF INTEREST STATEMENT

The authors declare no conflicts of interest.

## Supporting information


Figure S1

Figure S2

Data S1

## Data Availability

Data and code are available on Figshare: https://doi.org/10.6084/m9.figshare.24233548.v1.
