# Supplementary figures and images for: Generalized nonlinearity in animal ecology: Research, review, and recommendations
Source: Ecol Evol. 2024 Jul 11;14(7):e11387. doi: 10.1002/ece3.11387 (PMC11237342; doi:10.1002/ece3.11387)

**Coyote Relative Abundance GLM**

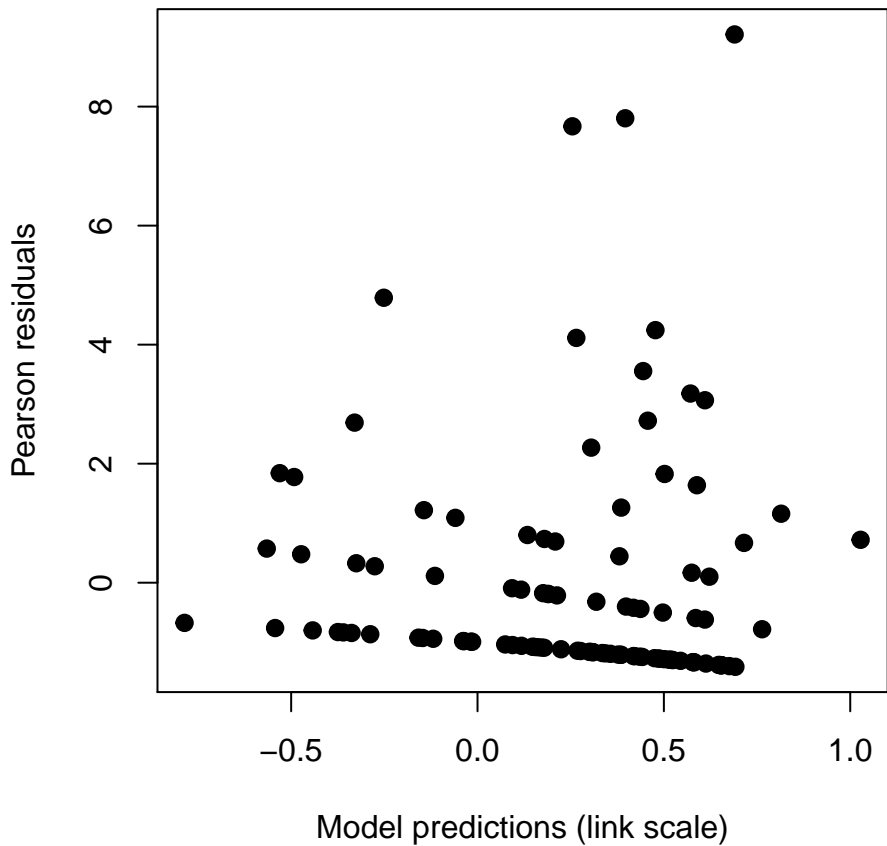

**Red Fox Relative Abundance GLM**

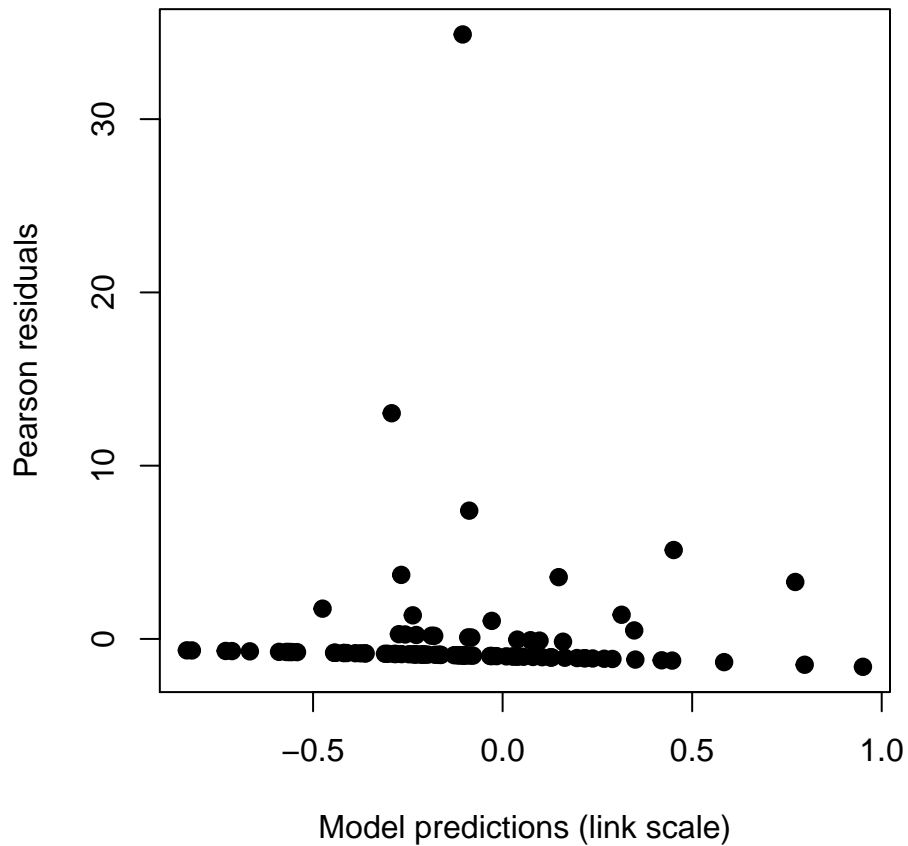

Supplement: Supplementary file 1 — Figure S1 [file ECE3-14-e11387-s003.pdf]

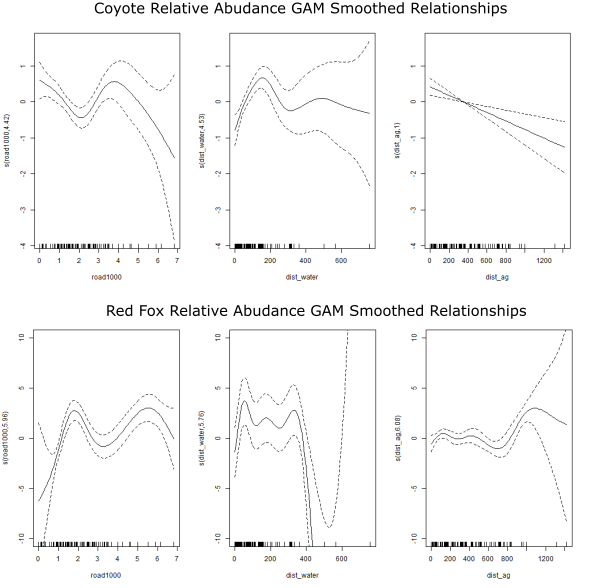

Supplement: Supplementary file 2 — Figure S2 [file ECE3-14-e11387-s001.png]
